# Naringin Alleviates H_2_O_2_-Inhibited Osteogenic Differentiation of Human Adipose-Derived Stromal Cells via Wnt/*β*-Catenin Signaling

**DOI:** 10.1155/2022/3126094

**Published:** 2022-04-29

**Authors:** Xufang Yang, Jianjiang Dong, Yankun Hao, Yucheng Qi, Jun Liang, Lei Yan, Wenting Wang

**Affiliations:** ^1^Department of Pathophysiology, Mudanjiang Medical University, Mudanjiang 157011, China; ^2^Department of Histology and Embryology, Mudanjiang Medical University, Mudanjiang 157011, China; ^3^Department of Medical Function, Mudanjiang Medical University, Mudanjiang 157011, China; ^4^Basic Medical College, Mudanjiang Medical University, Mudanjiang 157011, China; ^5^Stem Cell Institute, Mudanjiang Medical University, Mudanjiang 157011, China; ^6^Department of Physiology, Mudanjiang Medical University, Mudanjiang 157011, China

## Abstract

Osteoporosis is an age-related systemic bone disease that places a heavy burden on patients and society. In this study, we aimed to investigate the effects of naringin (NAR) on the osteogenic differentiation of human adipose-derived stromal cells (ADSCs). The results demonstrated that NAR pretreatment effectively abated H_2_O_2_-induced cell death and ROS accumulation in ADSCs undergoing osteogenic differentiation (ADSCs-OD). In addition, we also observed that the impaired extracellular matrix mineralization and ALP activity in H_2_O_2_-stimulated ADSCs-OD were notably rescued by NAR pretreatment. Moreover, the effects of H_2_O_2_ exposure on Wnt/*β*-catenin signaling in ADSCs-OD were largely reversed by NAR pretreatment. Collectively, our findings indicated that NAR could protect ADSCs-OD against H_2_O_2_-inhibited osteogenic differentiation.

## 1. Introduction

Osteoporosis is a common disease characterised by a systemic impairment of bone mass, strength, and microarchitecture which increases the propensity of fragility fractures [1]. Osteoporosis seriously affects patients' life quality and places a heavy burden on society. At the cellular level, this disease is caused by an imbalance between osteoclast-mediated bone resorption and osteoblast-mediated bone formation [2]. In 2001, adipose-derived stromal cells (ADSCs) were extracted for the first time by digestion of human adipose tissues [3]. ADSCs are abundant and can be easily acquired [4]. The in vitro and in vivo osteogenetic capability makes ADSCs a promising source of seed cells in bone tissue engineering [5].

Traditional Chinese medicines (TCMs) have long been used to prevent and treat osteoporosis. They have fewer adverse reactions and are more suitable for long-term use compared with chemically synthesized medicines [6]. Naringin (NAR), a bioflavonoid abundant in grapefruit and other related citrus fruit species, has numerous biological and pharmacological properties [7]. It has been shown to prevent ovariectomy-induced osteoporosis and promote osteoclasts apoptosis via mitochondria-mediated apoptosis pathway [8]. In this study, we aimed to investigate the effects of NAR on the osteogenic differentiation of human ADSCs.

## 2. Materials and Methods

### 2.1. Human Samples

The samples of human abdominal fat were obtained from 10 donors (the average age was 33.6 ± 4.8; 5 males, 5 females) who underwent liposuction at hospital. Patients with healthy physical examination results were included. Patients with malignant tumors, autoimmune diseases, congenital diseases, and genetic diseases were excluded. None of the participants had systemic diseases or infections. This study was approved by the ethics committee of the hospital, and written informed consent was obtained from all participants.

### 2.2. Isolation and Culture of ADSCs

The samples of human abdominal fat were washed twice with 10 ml phosphate-buffered saline (PBS) to remove blood and grease, digested with collagenase type I solution (Sigma-Aldrich, St. Louis, MO, USA) for 1 h at 37°C, and filtered through 250 *μ*m filters. Following centrifugation (500 × g, 5 min, 3×), the cells were collected and cultured in Dulbecco's modified Eagle's medium (DMEM; Invitrogen, Carlsbad, CA, USA) containing 10% fetal bovine serum (FBS; HyClone, Logan, UT, USA) and 1% penicillin/streptomycin at 37°C in a humidified incubator with 5% CO2. Cells at passage 3 were used in the following experiments.

### 2.3. Osteogenic Induction and NAR Treatment

To induce osteogenic differentiation, ADSCs seeded in 24‐well plates (1 × 104 cells/well) were treated with osteogenic medium (OM), which consisted of standard culture medium supplemented with 0.1 mM dexamethasone, 7 mM sodium *β*-glycerophosphate, and 200 *μ*M ascorbic acid. The medium was renewed every 2 d. After 14 d, cells were stained with Alizarin red S to confirm the existence of mineralized nodules.

The experiments were divided into four groups:

- ADSCs in Group 1 were cultured in OM for 14 d.
- After incubation in OM for 48 h, ADSCs in Group 2 were treated with 0.2 mM H_2_O_2_ for 4 h. The supernatant was then discarded, and ADSCs were further cultured in OM for 12 d.
- ADSCs in Group 3 were cultured in OM for 24 d. Then, 0.1 mM NAR was added to each well, and ADSCs were further cultured for 13 d.
- After incubation in OM for 24 h, ADSCs in Group 4 were treated with 0.1 mM NAR for 24 h. Then 0.2 mM H_2_O_2_ was added to each well. After additional 4 h, the supernatant was discarded, and ADSCs were further cultured in OM + 0.1 mM NAR for 12 d.

### 2.4. Alizarin Red S (ARS) Staining Assay

The cells were fixed in 4% paraformaldehyde, and stained with 0.1% ARS staining solution (pH 4.2; Sigma-Aldrich). After 1 h, the stained cells were observed by a light microscope (Olympus, Tokyo, Japan).

### 2.5. Alkaline Phosphatase (ALP) Staining Assay

The cells were fixed in 4% paraformaldehyde, and stained with BCIP/NBT working solution (Beyotime, Shanghai, China). After 0.5 h, the stained cells were observed by a light microscope.

### 2.6. Cell Viability Analysis

Cell viability was determined by the MTT assay. The cells were seeded into 96-well plates. After the aforementioned treatments, 20 *µ*l MTT solution (5 mg/l; Sigma-Aldrich) was added to each well. After additional 4 h, the supernatant was discarded, and 150 *µ*l DMSO was added to dissolve the violet formazan crystals. The absorbance was measured at 570 nm using a microplate reader (Molecular Devices, Sunnyvale, CA, USA).

### 2.7. Measurement of Intracellular ROS Accumulation

Intracellular ROS production was detected with DCFH-DA fluorescent probe (Sigma-Aldrich). The cells were incubated with 20 *µ*l DCFH-DA solution at 37°C for 30 min. The fluorescence intensity was measured by using a FACScan flow cytometer (BD Biosciences, Franklin lakes, NJ, USA).

### 2.8. RT-qPCR Analysis

Total RNA was extracted from cells using TRIzol reagent (Invitrogen), and then reverse-transcribed into cDNA by the PrimeScript RT reagent Kit (TaKaRa, Dalian, China). PCR amplifications were then carried out using a SYBR Green PCR Kit (TaKaRa) on a 7500 Fast Real-Time Sequence detection system (Applied Biosystems, Foster City, CA, USA). The data were analyzed using 2−ΔΔCt method [9], and *β*-actin was employed as an internal control.

### 2.9. Western Blot Analysis

Cell lysates were prepared with RIPA buffer (Beyotime). Identical quantity of protein samples were separated by SDS-polyacrylamide gels, and transferred to PVDF membranes (Millipore, Billerica, MA, USA). Following blocking in 5% nonfat milk for 1 h, the membranes were incubated with specific primary antibodies and HRP-conjugated secondary antibody. The protein bands were visualized by the Immobilon ECL substrate kit (Millipore), and *β*-actin was employed as an internal control.

### 2.10. Statistical Analysis

All statistical analyses were performed using the GraphPad Prism 6.0 software (GraphPad Software, Inc., La Jolla, CA, USA). Data were expressed as mean ± standard deviation (SD) of three repeated experiments. The significance of differences between groups was assessed by one-way analysis of variance followed by Tukey's test. Values of *p* < 0.05 were considered to indicate a statistically significant result.

## 3. Results

**Figure 1:**
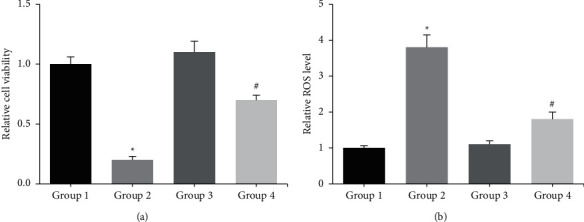
(a) The viability of ADSCs-OD was detected by MTT assay. (b) The intracellular ROS accumulation in ADSCs-OD was detected by DCFH-DA staining. ^*∗*^*p*  <  0.05 vs. Group 1; #*p* < 0.05 vs. Group 2.

We first confirmed that treatment with 0.1 mM NAR for 13 d did not have any significant effect on the survival of ADSCs undergoing osteogenic differentiation (ADSCs-OD), as determined by the MTT assay, but 4 h of exposure with 0.2 mM H_2_O_2_ notably reduced cell viability, and this toxic effect was markedly rescued by NAR pretreatment (Figure 1(a)). In addition, NAR pretreatment also dramatically reduced the H_2_O_2_-induced ROS accumulation in ADSCs-OD (Figure 1(b)).

**Figure 2:**
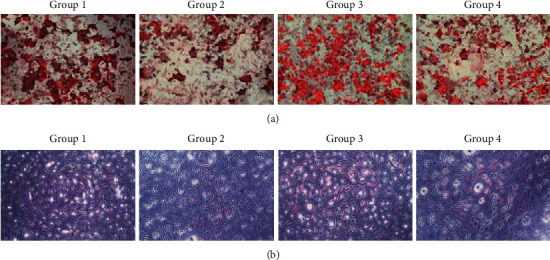
(a) The extracellular matrix mineralization in ADSCs-OD was detected by ARS staining. (b) The ALP activity in ADSCs-OD was detected by ALP staining.

Moreover, as shown in Figure 2, the impaired extracellular matrix mineralization and ALP activity in H_2_O_2_-stimulated ADSCs-OD were significantly blocked by NAR pretreatment.

**Figure 3:**
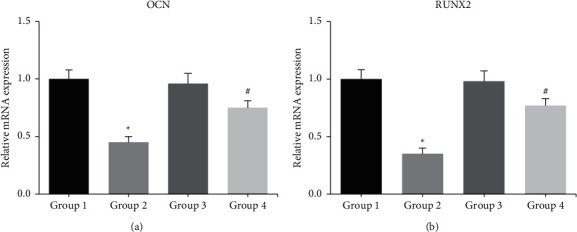
The mRNA levels of osteogenesis-related genes in ADSCs-OD were detected by RT-qPCR analysis. ^*∗*^*p* < 0.05 vs. Group 1; #*p* < 0.05 vs. Group 2.

Through RT-qPCR analysis, we noted that after H_2_O_2_ exposure, the mRNA levels of osteogenesis-related genes, such as OCN and RUNX2, were significantly reduced in ADSCs-OD, but these effects were obviously restored by NAR pretreatment (Figure 3).

**Figure 4:**
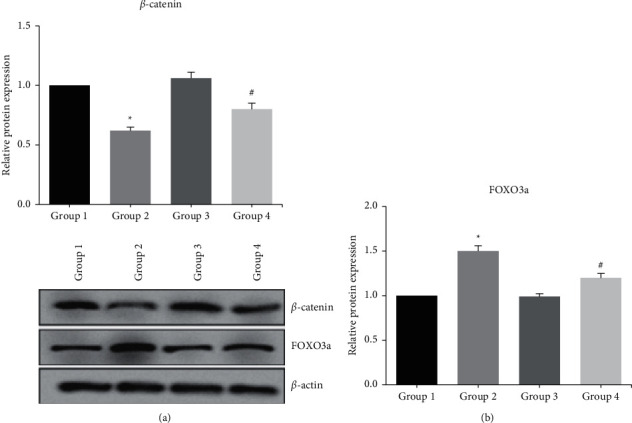
The expression levels of Wnt/*β*-catenin signaling-related proteins in ADSCs-OD were detected by western blot analysis. ^*∗*^*p* < 0.05 vs. Group 1; #*p* < 0.05 vs. Group 2.

Furthermore, through western blot analysis, we observed that the effects of H_2_O_2_ exposure on the expression levels of Wnt/*β*-catenin signaling-related proteins, such as *β*-catenin and FOXO3a, in ADSCs-OD were largely reversed by NAR pretreatment (Figure 4).

## 4. Discussion

Osteoblasts play a major role in bone formation. ADSCs can be differentiated into osteoblasts under specific induction, and improving the osteogenic differentiation ability of ADSCs is of critical importance for bone regeneration. Oxidative stress, resulting from excessive generation of reactive oxygen species (ROS), inhibits osteoblastic differentiation of bone cells [10]. This research used H_2_O_2_-induced oxidative stress model, and we confirmed that NAR pretreatment could reduce the oxidative damage caused by H_2_O_2_ in ADSCs-OD. The inhibition of osteoblastic differentiation caused by H_2_O_2_ was featured by the reduction of ALP activity, a critical regulator of bone matrix mineralization [11]. Patients with osteoporosis often have lower OCN and RUNX2 compared to healthy people [12]. This study further showed that H_2_O_2_-inhibited osteogenic differentiation was notably rescued by NAR pretreatment.

Wnt/*β*-catenin signaling is involved in the regulation of diverse pathophysiological processes, and it also plays a key role in osteogenesis by determining the differentiation of stem cells into mature osteoblasts rather than into chondrocytes and adipocytes [13]. Attenuation of Wnt/*β*-catenin signaling may be responsible for decreased bone formation and increased bone marrow adiposity [14]. Oxidative stress antagonizes Wnt/*β*-catenin signaling in osteoblast precursors by activating FOXO3a transcription factor [15]. The present study also verified that the effects of H_2_O_2_ exposure on Wnt/*β*-catenin signaling in ADSCs-OD were obviously reversed by NAR pretreatment.

In conclusion, our study provides promising evidence that NAR could protect ADSCs-OD against H_2_O_2_-inhibited osteogenic differentiation. Therefore, NAR may be a potential therapeutic approach for treating patients with osteoporosis.

## Data Availability

The data used to support the findings of this study are available from the corresponding author upon request.
